# NLRP3 Knockout Protects against Lung Injury Induced by Cerebral Ischemia–Reperfusion

**DOI:** 10.1155/2022/6260102

**Published:** 2022-04-08

**Authors:** Qingxue Xu, Yingze Ye, Zhuo Wang, Hua Zhu, Yina Li, Jin Wang, Wenwei Gao, Lijuan Gu

**Affiliations:** ^1^Central Laboratory, Renmin Hospital of Wuhan University, Wuhan 430060, China; ^2^Department of Anesthesiology, Renmin Hospital of Wuhan University, Wuhan 430060, China; ^3^Department of Neurosurgery, Renmin Hospital of Wuhan University, Wuhan 430060, China; ^4^Department of Critical Care Medicine, Renmin Hospital of Wuhan University, Wuhan 430060, China

## Abstract

**Methods:**

C57/BL6 wild-type (WT) and NLRP3-KO mice were used to construct middle cerebral artery occlusion (MCAO) models. 2,3,5-Triphenyltetrazolium chloride (TTC) was used to evaluate brain damage, and neurological deficits were assessed. Then, lung tissue injury was examined in the different groups of mice by hematoxylin-eosin (HE) staining. Inflammation (macrophage and neutrophil infiltration, NLRP3-associated inflammatory molecules) and oxidative stress (reactive oxygen species, ROS) in the lungs were comprehensively examined by immunofluorescence staining and Western blotting.

**Results:**

First, our findings demonstrated that NLRP3 knockout had a protective effect against cerebral ischemia–reperfusion injury after MCAO. Second, by reducing brain damage after MCAO, lung inflammation was also alleviated. Immunofluorescence staining showed that NLRP3-KO-MCAO mice had reduced inflammatory effector molecule (caspase-1 and IL-1*β*) expression and macrophage and neutrophil infiltration in the lung, as well as remissive oxidative stress state in the lung, compared with WT-MCAO mice. We also observed a decrease in phosphorylated p65 (p-p65) (an NF-*κ*B factor) in NLRP3-KO-MCAO mice, suggesting that the NF-*κ*B pathway was involved in the protective effect of NLRP3 gene knockout on stroke-induced lung injury.

**Conclusions:**

NLRP3 inflammasome knockout not only is beneficial for cerebral ischemia–reperfusion injury but also reduces the severity of poststroke lung injury by reducing brain damage. It has been confirmed that there is a relationship between central insult and peripheral organ injury, and protecting the brain can prevent peripheral organ damage.

## 1. Introduction

Ischemic stroke is a disease with high mortality and disability rates that are caused by a temporary or permanent reduction in local blood flow. In addition to death from ischemic stroke itself, peripheral organ damage, such as stroke-associated pneumonia (SAP) and urinary tract infection (UTI), can also occur [1, 2] because the respiratory and urinary systems are the most susceptible.

The inflammatory response plays an integral role in stroke and SAP [3]. The acute immune response after stroke leads to immediate brain tissue damage, and early microglial activation leads to increased blood–brain barrier (BBB) permeability and circulating neutrophil and lymphocyte infiltration [4, 5]. Immune cells release cytokines and chemokines (such as TNF-*α*, IL-1*β*, IL-17, and IL-23) that exacerbate inflammation and increase infarct size after stroke [6, 7]. Recruitment of circulating leukocytes to the damaged brain results in a reduction in peripheral immune cells, which is one of the reasons for peripheral immunosuppression. In addition, brain injury leads to robust activation of the sympathetic nervous system (SNS), which also causes robust peripheral immunosuppression and leads to an increased incidence of poststroke infection [8–10]. Peripheral immunosuppression after stroke manifests as increased lymphocyte apoptosis, decreased lymphocyte numbers, and splenic atrophy. In addition, activation of the hypothalamic–pituitary–adrenal (HPA) axis after increases glucocorticoid release through endocrine signaling, resulting in the stagnation of B lymphocyte production and a decrease in peripheral B lymphocytes [11]. Therefore, stroke-induced immunosuppression increases the risk of lung infection, and stroke can also lead to neurogenic lung injury. Acute lung injury (ALI) is a common complication after stroke that clinically manifests as varying degrees of dyspnea, breathlessness, and cyanosis. Stroke-induced ALI is related to pulmonary inflammation and a decrease in cellular immune function. Studies have shown that increased concentrations of proinflammatory cytokines in bronchoalveolar lavage fluid (BALF) were found in patients with fatal cerebral damage [12]. In animal studies, whole-lung proinflammatory cytokines are increased after ischemic stroke, causing significant BALF inflammation (increased macrophages and neutrophils) and significant diffuse alveolar injury and pulmonary edema in the lungs [11, 13]. NLRP3 is an important component of the inflammatory response that is activated by damage-associated molecular patterns (DAMPs), such as reactive oxygen species (ROS), K^+^, Ca^2+^, and acids released by damaged tissues after stroke [14], and subsequently mediates caspase-1 activation to cleave and produce mature IL-1*β* and IL-18, ultimately leading to neuroinflammation or pyroptosis [15]. NLRP3 is widely involved in aseptic inflammation in a variety of organs throughout the body, and NLRP3 activation plays a major role in the progression of brain and lung tissue injury [16].

However, although a connection between the brain and the lung exists, there is no evidence to show whether pulmonary inflammation is reduced after measures to protect against cerebral ischemia–reperfusion injury (CIRI). In addition, it is still unknown whether inhibiting NLRP3, a key molecule involved in brain inflammation, reduces the degree of lung injury after the improvement of brain injury. In this study, we determined the effect of NLRP3 knockout (KO) on ischemic brain and lung injury resulting from stroke by using a middle cerebral artery occlusion (MCAO) focal ischemia mouse model and attempted to further clarify the crosstalk between cerebral ischemia and ALI.

## 2. Materials and Methods

### 2.1. Animals

Wild-type (WT) C57BL/6 J mice (aged 8-10 weeks, 22–25 g, *n* = 160; unsuccessful dead and unqualified animals were excluded, success rate of approximately 70%) were purchased from Henan Skibis Biological Technology Co., Ltd. (Henan, China; nos. 410983211100088868 and 410983211100122821). NLRP3-KO mice were donated by the Department of Cardiology, Renmin Hospital of Wuhan University. The mice (aged 8-10 weeks) used in this study were further bred in the Central Laboratory of Renmin Hospital of Wuhan University.

All animal experimental protocols were approved by the Animal Experimentation Ethics Committee of Wuhan University (no. WDRM-20170504). Animals were housed in the Animal Experimental Center of Renmin Hospital of Wuhan University with free access to food and water for at least 1 week before the experiments.

### 2.2. MCAO Model

Mice were anesthetized with 5% isoflurane in O2 with a facemask, and the intraoperative oxygen flow was maintained at 4-6 L/min with 1-1.5% isoflurane. The left internal carotid artery was exposed, and a 6-0 monofilament (Doccol, Corp., Redlands, CA, United States) was inserted to ligate the left arteria carotis communis and external carotid arteries. The cephalic artery was opened cephalically, and the suture was inserted along the internal carotid artery. After 1 hour of occlusion, the suture was removed, and reperfusion was performed. A thermostatic heating pad was used to monitor and stabilize the body temperature of the mice at 37 ± 0.5°C. There was no thrombus inserted in the Sham group.

### 2.3. Infarct Volume Measurement

Mice were deeply anesthetized and euthanized with an overdose of chloral hydrate. The mice were sacrificed, and the brain was collected 3 days after ischemia–reperfusion (I/R). After normal saline was perfused into the left ventricle, brain tissue was collected for protein extraction. After being perfused with 4% paraformaldehyde (PFA), brain tissue was fixed for immunofluorescence staining. Then, after being stained with 2% 2,3,5-triphenyltetrazolium chloride (TTC) (17779, Sigma–Aldrich, USA), the cells were fixed with PFA for 24 h and scanned, and the infarct volume was calculated with ImageJ software. To exclude the influence of cerebral edema on the infarcted side of the brain, the formula [(area of noninfarcted hemisphere − area of infarcted hemisphere)/area of noninfarcted hemisphere] × 100% was used to calculate the hemispheric infarct ratio and assess the degree of cerebral infarction.

### 2.4. Assessment of Neurological Deficits

Neurological deficits were scored by single-blinded observers 3 days after surgery using the Longa score, which ranged from 0 (no significant neurological deficits) to 4 (no spontaneous motor activity or loss of consciousness). When activity was normal, and there was no neurological defect, the score was 0. When the right (the opposite side of the infarcted hemisphere) forelimb was not fully expanded and nerve function was slightly deficient, the score was 1. When the mice turned to the right when walking with moderate neurological deficits, the score was 2. When the mouse walked, the body fell to the right, and there was severe neurological impairment; the score was 3. When there was an inability to walk spontaneously with conscious disturbance, the score was 4.

### 2.5. Immunohistochemical Staining

Lung tissue was perfused with 4% PFA, embedded in paraffin, and then sectioned. The thickness was 3-4 *μ*m, and the specimen was installed on a slide. After hematoxylin-eosin staining (HE), all tissue sections were examined under an optical microscope (Olympus Optical Ltd., Tokyo, Japan). The degree of edema and inflammation in lung tissue was assessed, and pathological analysis was performed.

### 2.6. Immunofluorescence Staining

First, the mice were euthanized and infused with phosphate-buffered saline PBS (0.01 M, pH 7.40, 4°C) and then infused with 4% PFA (pH 7.40, 4°C). The brain was fixed by being immersed in the same PFA solution (48 hours, 4°C). Some lung samples were embedded in paraffin, and others were embedded, frozen (-20°C), cut into 4 *μ*m coronal sections, and installed on slides. The paraffin slides were stained after the slices were washed with PBS. The slices were blocked with 0.1 M PBS containing 5% fetal bovine serum and 0.3% Triton X-100 for 1 h at room temperature. After being washed with PBS, primary antibody was added and incubated overnight (12 hours, 4°C). Then, the corresponding secondary antibody was added and incubated (2 hours, 25°C). The slides were cleaned and sealed with DAPI reagent, followed by automatic fluorescence microscopy (BX63, Olympus Optical, Ltd., Tokyo, Japan) observation. Frozen-slide ROS dye solution (1 : 500; D7008, Sigma, USA) was used, and immunofluorescence analysis was performed after the slides were sealed and fixed. The number of immunoreactive cells was counted using ImageJ software (Media Cybernetics, Inc., Rockville, MD, USA). Four different fields for each mouse and three mice for each group were counted. The data were analyzed by single-blind observers. The primary antibodies were anti-NLRP3 (1 : 200; ab4207, Cell Signaling Technology, Boston, MA, USA), anti-NeuN (1 : 200; ab104224, Abcam, Cambridge, UK), anti-MPO (1 : 100, GB11224, Servicebio, Wuhan, China), anti-CD68 (1 : 200; MCA1957, AbD Serotec, Oxford, UK), anti-CL-caspase-1 (1 : 50; 89332, Cell Signaling Technology, Boston, MA, USA), and anti-IL-1*β* (1 : 200; Abcam, Cambridge, UK). The secondary antibodies were Alexa 594-conjugated (1 : 200 for Casepase-1, CD68 and MPO; ANT030, Millipore, Billerica, MA, USA) or Alexa 488-conjugated (1 : 200 for IL-1*β*; ANT024, Millipore, Billerica, MA, USA).

### 2.7. Western Blot Analysis

The left hemisphere was weighed and lysed in RIPA lysis buffer (tissue (g): RIPA buffer = 1 : 10). Centrifugation was performed at 12000 rpm for 15 min at 4°C, and the concentration of the supernatant was normalized. The protein solution was mixed with loading buffer and boiled at 100°C for 10 min to denature the proteins. The protein sample (10-20 *μ*L) was loaded into sodium dodecyl sulfate-polyacrylamide gels (8%-15%) for electrophoresis. After protein separation, the gel was transferred to a PVDF membrane (Millipore, Billerica, MA, USA). Then, the PVDF membrane was washed with Tris-buffered saline containing Tween-20 (TBST, pH 7.40) and sealed in 5% BSA solution for 1 hour. Primary antibodies were diluted with primary antibody diluent (AS1061, Aspen) according to the manufacturer's instructions and then incubated overnight (12 hours, 4°C). After incubation, the membrane was washed with TBST, and the secondary antibody was added and incubated (2 hours, 25°C). Then, the membranes were washed with TBST, and images were acquired with an Odyssey Western Blot Analysis System (LI-COR, Lincoln, NE, USA). The primary antibodies included mouse anti-NLRP3 (1 : 1,000; ab4207, Cell Signaling Technology, Boston, MA, USA), anti-ASC (1 : 1,000; 67824, Cell Signaling Technology, Boston, MA, USA), anti-CL-caspase-1 (1 : 500; 89332, Cell Signaling Technology, Boston, MA, USA), anti-IL-1*β* (1 : 500; ab8320, Abcam, Cambridge, UK), anti-IL-18 (1 : 500; ab207324, Abcam, Cambridge, UK), anti-NF-*κ*B p65 (1 : 1,000; ab32536, Abcam, Cambridge, UK), antiphosphorylated NF-*κ*B p65 (1 : 1,000; 3033S, Cell Signaling Technology, Boston, MA, USA), anti-*β*-tubulin (1 : 200, GB11017B, Servicebio, Wuhan, China), and anti-GAPDH (1 : 2,000; GB11002, Servicebio, Wuhan, China). The secondary antibodies included IRDye-labeled anti-rabbit (1 : 10,000; 926-32211, Li-Cor Bioscience, USA) and IRDye-labeled anti-mouse (1 : 10,000; 926-32210, Li-Cor Bioscience, USA) secondary antibodies.

### 2.8. Measurement of GSH Levels

Fresh brain tissues were collected 3 days after MCAO, and GSH levels were measured with a commercial GSH assay kit (Nanjing Jiancheng, Nanjing, China). All steps were performed in strict accordance with the instructions.

### 2.9. Statistical Analysis

GraphPad Prism 8 and SPSS 26.0 statistical software were used. All experimental data are presented as the mean ± SEM, and a completely randomized design of one-way analysis of variance (ANOVA) was used for comparisons between groups. Homogeneity of variance was tested by the LSD-T test. When *P* < 0.05, there was a significant difference.

## 3. Results

### 3.1. NLRP3 KO Protected against CIRI after MCAO

**Figure 1:**
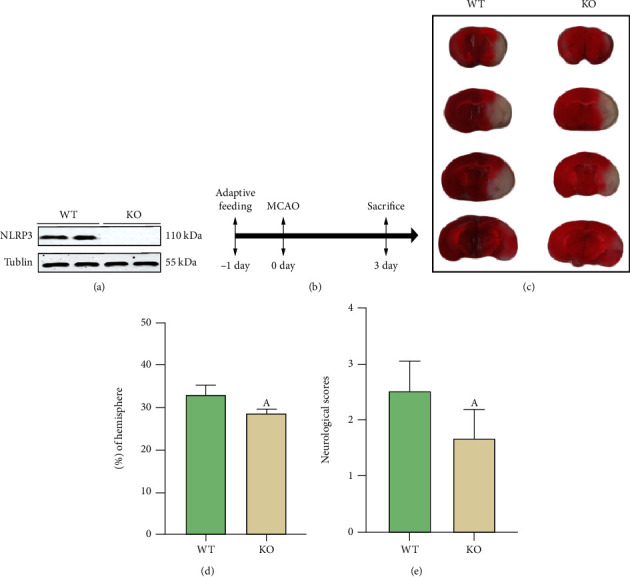
NLRP3-KO protected the cerebral ischemia-reperfusion injury (CICR) induced by MCAO. (a) Western blot shows the protein expression of NLRP3. (b) MCAO model construction and experimental design. (c) TTC result is shown to assess the infarction size. (d) Infarct volume was normalized to the contralateral hemicerebrum and presented as a percentage. (e) Neurological scores. The data are presented as the mean ± SEM. *n* = 5 per group. ^A^*P* < 0.05 vs. WT.

The NLRP3 inflammasome is a key factor in the progression of CIRI. We wanted to determine whether there as a protective effect of NLRP3 KO on the brain after CIRI. Therefore, 72 hours post-MCAO, neurofunctional assessment was evaluated, and the mice were sacrificed to collect brain tissue to measure the cerebral infarction volume (Figure 1(b)). We first confirmed by Western blotting that NLRP3 protein was not expressed in NLRP3-KO mice (Figure 1(a)) and then found that the cerebral infarct volume of NLRP3-KO mice was significantly reduced compared with that of wild-type (WT) mice (Figures 1(c) and 1(d)), and the neurobehavioral scores of NLRP3-KO mice are significantly lower (Figure 1(e)). This result indicated that NLRP3 gene KO alleviated CIRI in MCAO mice.

### 3.2. NLRP3 KO Relieved Inflammation in the Lungs by Reducing Brain Damage after MCAO

**Figure 2:**
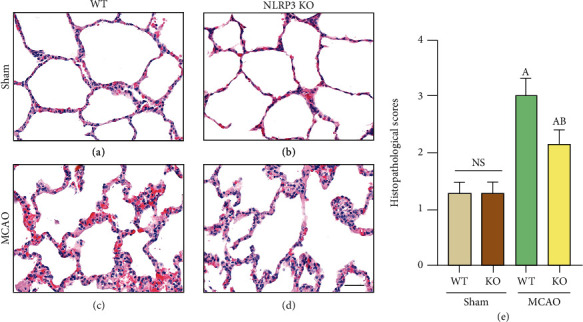
NLRP3-KO protected pulmonary inflammation induced by MCAO. (a)–(e) HE staining is showing the lung tissue injury after Sham surgery (a, b) and MCAO (c, d) in WT mice and NLRP3-KO mice. (d) Histopathological score of HE staining. The data are presented as the mean ± SEM. *n* = 5 per group. Scale bar = 50 *μ*m. ^A^*P* < 0.05 vs. Sham; ^B^*P* < 0.05 vs. WT.

Recent studies have shown that SAP, which causes lung injury, is a common complication after stroke. We compared the lung tissues of WT-Sham, WT-MCAO, NLRP3-KO-Sham, and NLRP3-KO-MCAO mice by HE staining. First, the results showed that NLRP3-KO mice had no significant inflammatory response or lung injury compared with WT mice in the Sham group (Figures 2(a), 2(b), and 2(e)). MCAO resulted in significant thickening of the alveolar wall and marked infiltration of proinflammatory cells, hemorrhage, and inflammatory responses in WT mice (Figures 2(a), 2(c), and 2(e)). Among NLRP3-KO mice, those in the MCAO group and Sham group also had the same trend as WT mice, which indicated that lung injury was aggravated after MCAO (Figures 2(b), 2(d), and 2(e)). However, when comparing NLRP3-KO-MCAO and WT-MCAO mice, lung injury was relieved, and mild lung inflammation occurred in the NLRP3-KO group, while severe lung inflammation was present in WT-MCAO mice (Figures 2(c), 2(d), and 2(e)). These results confirm that stroke can cause pneumonic injury and preliminarily indicate that NLRP3 KO not only protects against ischemic brain injury but also improves stroke-induced lung inflammation.

### 3.3. NLRP3 KO in Mice Reduced Lung Inflammasome-Related Protein Expression in the Lung

**Figure 3:**
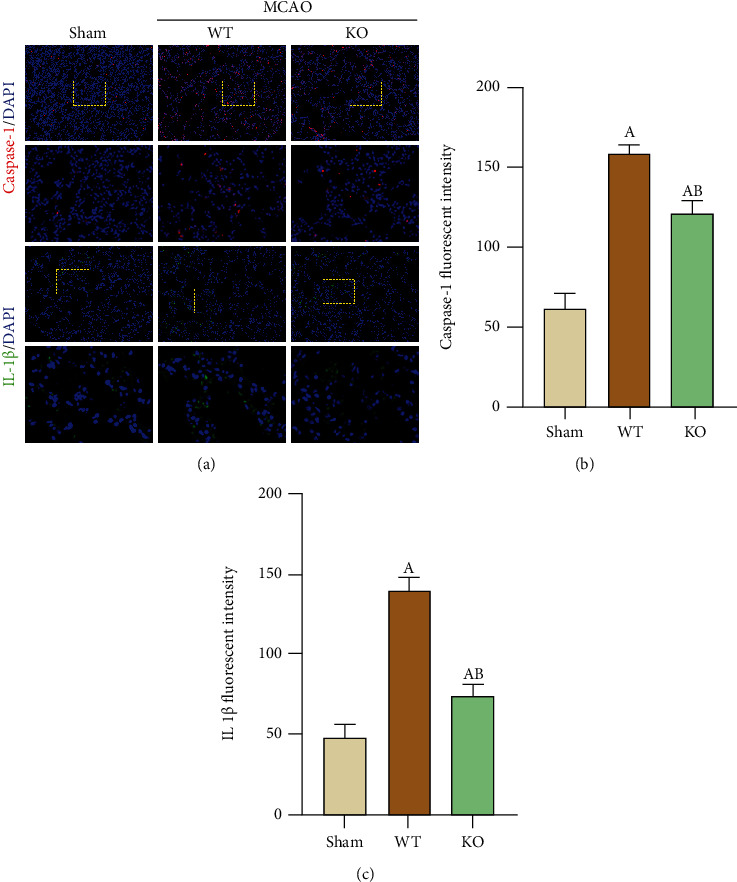
NLRP3-KO inhibited the expression of inflammasome-related proteins in the lung after 3 days of MCAO. (a) Representative immunofluorescence staining to show the expression levels of Caspase-1 (red) and IL-1*β* (green), counterstained with DAPI (blue). Fluorescence density analysis of (b) Caspase-1 and (c) IL-1*β*. The data are presented as the mean ± SEM. *n* = 5 per group. Scale bar = 50 *μ*m. ^A^*P* < 0.05 vs. Sham; ^B^*P* < 0.05 vs. WT.

Caspase-1 is a downstream molecule of NLRP3, and IL-1*β* is an effector molecule that is released after caspase-1 is cleaved and matured [17]. After stroke, NLRP3-mediated inflammation is also activated in lung tissue. Compared with those in the WT-Sham group, the expression of caspase-1 and IL-1*β* levels was increased in the lungs of the WT-MCAO group by using immunofluorecence staining, while decreased in the NLRP3-KO-MCAO group (Figure 3). Our study demonstrated that after NLRP3 KO, NLRP3-related proteins were downregulated, since lung NLRP3 was reduced and lung damage resulting from the relief of brain damage was alleviated, demonstrating that neuroinflammation caused by stroke can adversely affect the lungs.

### 3.4. NLRP3 KO Mitigated Infiltration of Macrophage and Neutrophil in the Lung

**Figure 4:**
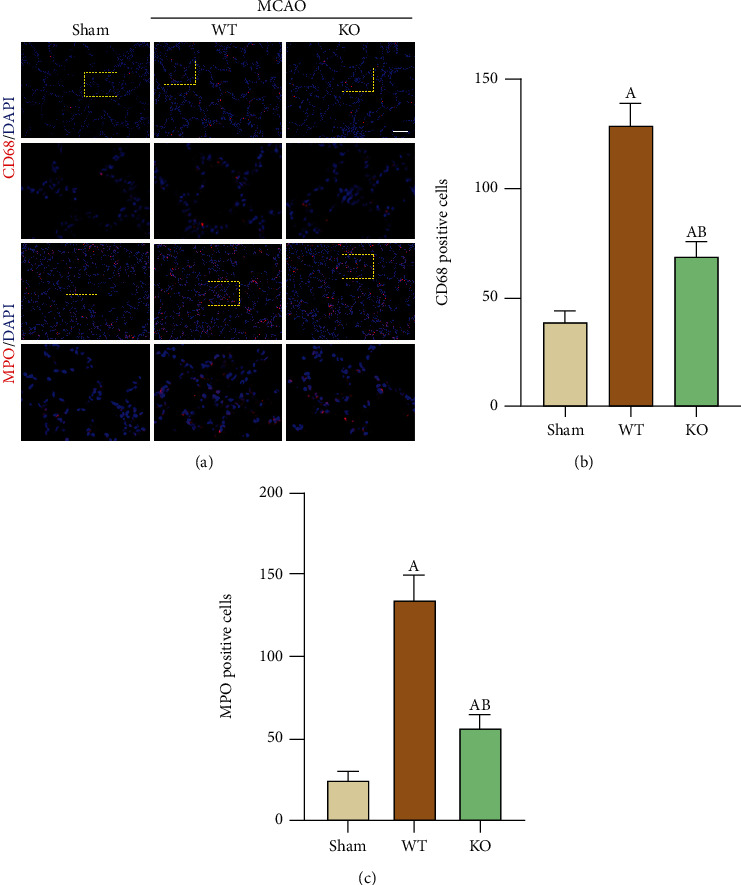
NLRP3-KO reduced the infiltration of macrophages and neutrophils in the lung after 3 days of MCAO. (a) Immunofluorescence was used to detect infiltration of macrophages (CD68^+^) and neutrophils (MPO^+^) in the injured lung following 3 days of MCAO, and representative images are showing. (b, c) Quantification of CD68 and MPO positive cells in the selected region, respectively. The data are presented as the mean ± SEM. *n* = 5 per group. Scale bar = 50 *μ*m. ^A^*P* < 0.05 vs. Sham; ^B^*P* < 0.05 vs. WT.

It is generally believed that the SNS and parasympathetic nervous system are activated after stroke, leading to peripheral immunosuppression and decreased immune function; thus, peripheral organs such as the lung and kidney are prone to infection. We observed alteration of lung tissue by immunofluorescence staining, and compared with mice in the WT-Sham group, WT-MCAO mice had more macrophage and neutrophil infiltration in the lung interstitium, while the number of inflammatory cells in NLRP3-KO-MCAO mice was reduced compared with WT-MCAO mice (Figure 4). This finding suggests that preventing brain injury by NLRP3-KO also increases tolerance to peripheral infection and reduces the degree of stroke-induced lung injury.

### 3.5. NLRP3 KO Alleviates Oxidative Stress in the Lungs after Brain Injury

**Figure 5:**
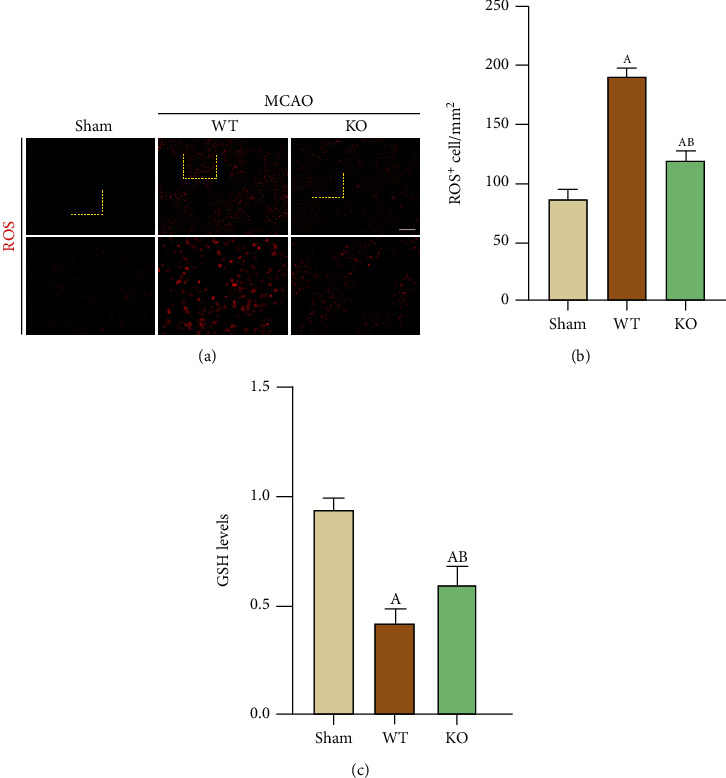
NLRP3-KO alleviated oxidative stress in the lung after 3 days of MCAO. (a) Representative ROS staining results of frozen sections of lung tissue. (b) Quantification of ROS positive cells. (c) Quantification of GSH level in fresh lung tissue by a GSH assay kit. The data are presented as the mean ± SEM. *n* = 5 per group. Scale bar = 50 *μ*m. ^A^*P* < 0.05 vs. Sham; ^B^*P* < 0.05 vs. WT.

We wanted to know whether the oxidative stress response in the lung paralleled inflammatory cell infiltration after lung injury induced by MCAO. We used immunofluoresent staining to detect the changes of ROS in frozen lung sections and commercial kit to assess the GSH level in the fresh lung tissue. The results showed that ROS levels in lung tissues in the WT-MCAO group were significantly increased, while GSH was decreased, than those of WT-Sham mice, NLRP3-KO significantly reversed the elevation of ROS and reduction of GSH resulted from MCAO, suggesting NLRP3 gene KO played a significant role in alleviating oxidative stress (Figure 5).

### 3.6. NF-*κ*B Pathway Participated in the Inflammatory Response of Stroke Associated Lung Injury

**Figure 6:**
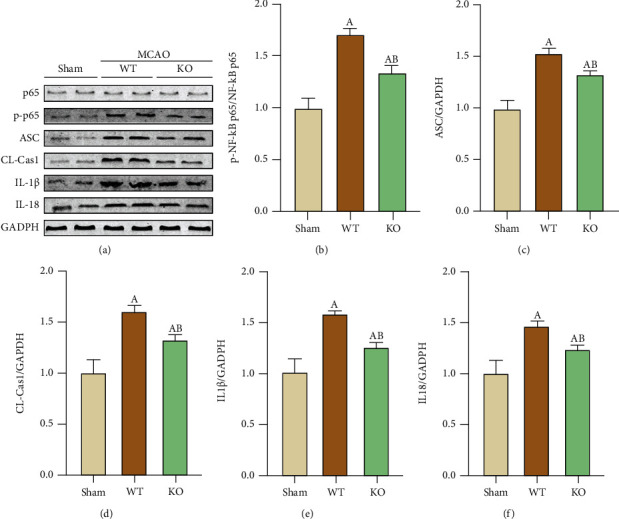
NF-*κ*B signaling pathway was involved in the ameliorative effect of NLRP3-KO on lung injury. (a) Representative western blot bands for p65, p-p65, ASC, CL-Cas1, IL-1*β*, and IL-18. (b ~ f) Quantification of ASC/GAPDH, CL-Cas1/GAPDH, IL-1*β*/GAPDH, IL-18/GAPDH, and p-p65/GAPDH, respectively. The data are presented as the mean ± SEM. *n* = 5 per group. ^A^*P* < 0.05 vs. Sham; ^B^*P* < 0.05 vs. WT.

We then used Western blotting to analyze ASC, the NLRP3 downstream molecule caspase-1, and the effector molecules IL-1*β* and IL-18 in the lung tissues of the different groups, and the results showed that the expression of these proteins was consistent with the immunofluorescence staining results (Figures 6(a) and 6(c)–6(f)). In addition, we examined the expression of the NF-*κ*B pathway-related protein p65 and phosphorylated p65 (p-p65), and the results showed that there was no difference in p65 among the groups, while the p-p65, activation status of p65, was altered, which was strongly activated in WT-MCAO group, NLRP3-KO inhibited the p65 activation significantly (Figures 6(a) and 6(b)). The results showed that the trend of NF-*κ*B pathway activation is paralleled with the NLRP3 related proteins upregulation, which indicates that NF-*κ*B pathway is not only involved in inflammation after lung injury resulted from cerebral ischemia.

## 4. Discussion

Studies have shown that NLRP3 is involved in inflammatory injury in multiple organs. Our study showed that when NLRP3 KO improved brain damage, lung damage was also reduced. In this study, we first showed that NLRP3-KO mice were significantly protected against brain injury after MCAO by TTC staining. Thus, lung tissues were extracted to examine corresponding indicators. After MCAO, NLRP3-KO mice were compared with WT mice, and the expression of caspase-1, IL-1*β*, and IL-18, macrophage and neutrophil infiltration, and oxidative stress levels in the lungs were significantly decreased, while GSH levels were increased. We also observed an increase in p-p65 after MCAO and in NLRP3-KO mice, suggesting that the NF-*κ*B pathway is involved in the inhibition of stroke-induced lung injury. Our findings proved that NLRP3 KO not only protects against CIRI but also prevents lung injury induced by stroke.

NLRP3 inflammasome-activated caspase-1 is thought to cause pyroptosis by acting on gasdermin D (GSDMD) [18, 19]. Since NLRP3 KO removes a damaging pathway, NLRP3 KO mice have less brain damage than WT mice, which we did demonstrate. Thus, we examined the lung tissue and showed that lung injury was reduced. Other studies have shown that pulmonary NLRP3 deficiency protects against lung injury by reducing alveolar epithelial apoptosis and neutrophil accumulation [20]. In addition, previous studies have shown that glial maturation factor *β* (GMFB) in astrocytes is upregulated after cerebral ischemia, and this brain-derived GMFB can destroy pulmonary microvascular endothelial cells (PMVECs) by increasing ROS [21]. In our study, we compared the NLRP3-KO-MCAO and WT-MCAO groups and found that after NLRP3 KO, decreases in the downstream molecules of NLRP3 (caspase-1, IL-1*β*, and IL-18), a reduction in the cerebral infarction volume and the alleviation of MCAO-induced pneumonia were observed, including edema of the lung interstitium and the infiltration of inflammatory cells in the lung interstitium, suggesting that in addition to the protective effect of NLRP3 KO on lung tissue, improvements in brain injury also protected lung tissue. Therefore, in NLRP3-KO mice, the degree of lung injury was reduced with the improvement of stroke, which may be due to two reasons. First, NLRP3 was knocked out in the lung by systemic NLRP3 KO, resulting in a protective effect and reducing lung inflammation. Second, due to the improvement in brain injury, the activation of the autonomic nerve and HPA axis was reduced, and peripheral immunosuppression was improved. Furthermore, when the lungs are attacked by pulmonary pathogens, the damage is correspondingly reduced due to the relative enhancement of immunity. In both the WT and NLRP3-KO groups, MCAO induced lung inflammation. This result makes the link between stroke and pneumonia more definitive. Importantly, pneumonia still occurred in mice after NLRP3 KO, suggesting that poststroke pneumonia primarily depends on peripheral immunosuppression rather than NLRP3 activation in the lung itself.

Studies have shown that ROS are elevated after lung injury and that GSH is consumed due to oxidative stress activation [22]. Oxidative stress in the lungs can be reflected by ROS and GSH. ROS production in alveolar macrophages can be activated and worsen lung injury when the lungs are exposed to pathogens or other stimuli that are inhaled [23, 24]. ROS are also believed to mediate the activation of the NLRP3 inflammasome, which plays an important role in lung injury [25]. Conversely, activation of the NLRP3 inflammasome and the infiltration of inflammatory cells in tissues also promote ROS production [26]. Therefore, by inhibiting ROS, lung injury can be improved [27, 28]. Moreover, we measured ROS and GSH levels to examine the oxidative stress state of the lungs after stimulation. It is thought that when the peripheral immune system is suppressed, pathogens that would not cause pneumonia are more likely to do so. Therefore, when brain injury is alleviated, immune suppression is partially relieved, and the body's resistance is enhanced; thus, lung injury is also alleviated. In this study, when NLRP3 KO alleviated brain injury, lung ROS levels were reduced and GSH was increased, suggesting that the degree of oxidative stress in the lungs was reduced. We think one reason for the inhibition of oxidative stress might be the result of lung injury reduction following the alleviation of brain damage, and the other reason may be due to NLRP3 deficiency in the lung. Despite oxidative stress, inflammation is increased in stroke-induced lung injury, which is characterized by macrophage and neutrophil infiltration in the lung parenchyma. Studies have shown that macrophages produce inflammatory cytokines, such as IL-6, that cause lung damage [27]. However, after KO of the NLRP3 inflammasome, macrophage and neutrophil infiltration was inhibited, and the production of proinflammatory cytokines was reduced, which was also beneficial for reducing lung injury.

NF-*κ*B is an important intracellular signaling pathway in innate and acquired immunity and is involved in proinflammatory gene transcription. The p65 subunit is a key factor of NF-*κ*B. p65 is phosphorylated to an active state, and p-p65 is increased when the NF-*κ*B pathway is activated [29]. Studies have shown that the sulfylation of p65 or drug inhibition of p65 phosphorylation can inhibit inflammation [30, 31]. Therefore, we examined p-p65 changes to examine activation of the NF-*κ*B pathway. Our findings suggest that the NF-*κ*B pathway is involved in lung tissue damage after stroke. P-p65 was significantly increased in the WT group after MCAO, and activation of the NF-*κ*B pathway in lung tissue was also inhibited after the degree of lung injury was relieved following a reduction in cerebral infarct volume.

Studies in mice have shown that prophylactic protection against stroke by KO of NLRP3 improves brain damage, but whether NLRP3 alleviates peripheral organ injury induced by stroke is still unknown. Our study showed that the expression of inflammatory-related proteins, the infiltration of inflammatory cells, and the level of oxidative stress were significantly improved in the lung after protection of the ischemic brain. We showed that NLRP3 KO not only has a protective effect against CIRI but also improves lung injury. Through our results and previous studies, we hypothesized that this phenomenon might be due to improvements in peripheral immunosuppression after stroke remission, thus reducing infection in peripheral organs. However, how peripheral immunity is changed and the precise mechanisms by which cerebral ischemia affects peripheral immunity and organs remain to be investigated. Even so, this study provides ideas for further research on the interaction between the central nervous system and peripheral organs and provides inspiration for the clinical treatment of SAP.

## 5. Conclusion

Our study first proved that NLRP3 KO has a protective effect against CIRI after MCAO and then proved that KO also effectively alleviates lung injury induced by ischemic stroke and prevents SAP. However, the specific mechanism by which brain injury affects peripheral organs is still unclear and needs to be further studied.

## Data Availability

The figures used to support the findings of this study are included within the article.
